# Capacitive Insect Sensing Under a Single Dual-Arc Geometry: A Laboratory Benchmark of Four CDC Architectures

**DOI:** 10.3390/s26113306

**Published:** 2026-05-22

**Authors:** Sen-Miao Chen, Yu-Bing Huang, Jen-Cheng Wang, Joe-Air Jiang

**Affiliations:** 1 Department of Biomechatronics Engineering, National Taiwan University, Taipei 10617, Taiwan; d00631009@ntu.edu.tw; 2 Applied Zoology Division, Taiwan Agricultural Research Institute, Ministry of Agriculture, Taichung 413008, Taiwan; ybhuang@tari.gov.tw; 3 Department of Computer Science, National Taipei University of Education, Taipei 106, Taiwan; jcwang@mail.ntue.edu.tw; 4 Department of Medical Research, China Medical University Hospital, China Medical University, Taichung 40402, Taiwan

**Keywords:** capacitive sensing, adult terrestrial insect monitoring, single-geometry laboratory benchmark, CDC architecture comparison, body-size scaling, humidity robustness

## Abstract

Capacitive sensing offers a low-power, non-optical route for automated insect monitoring, but architecture-level benchmarking under shared geometry remains limited. Rather than presenting a general framework, this study proposed a configuration-specific laboratory benchmark comparing four sigma-delta and charge-transfers in a 6 mm dual-arc conduit at 25 °C, targeting six adult terrestrial arthropod species spanning a 25-fold range of the body cross-sectional area. Static measurements showed a strong linear relationship between ΔC_static and body cross-sectional area (17.96 fF/mm^2^, r = 0.995), supporting first-pass conduit sizing and detectability screening. In contrast, transit amplitudes were not monotonic with body size because posture, motion, and gap occupancy affected waveform shape. Under chamber conditions, static sensitivity degraded by less than 3.2% across all architectures from RH 40% to 80%. However, under the deployment-oriented noise model, SNR_FR degradation was substantially higher for charge-transfer devices (64.8–66.8%) than for Σ–Δ devices (≤35.5%), because the composite noise floor amplifies the effect of humidity-induced baseline drift. These results generated a conduit-specific reference dataset for preliminary capacitance-to-digital converter (CDC) selection within the tested 6 mm dual-arc geometry. In addition, the experimental validation focused on laboratory baseline noise characterization, long-term drift, and trap-integrated testing in temperature-controlled environments and natural-locomotion trials, providing critical information on configuration-specific architectures and body-size-scaling reference. This study serves as an initial step toward real-world capacitive insect sensing. Future studies will investigate additional conduit geometries and insect species to improve the robustness of the proposed framework.

## 1. Introduction

### 1.1. Research Background

The timeliness of pest detection data significantly affects control decisions and crop yield. Field monitoring still relies primarily on manual inspection, which is constrained by labor availability and coverage area; early infestation symptoms are often recorded only after they become visible to the naked eye. As sensor and communication component costs have continued to decline, automated pest monitoring systems have progressed from concept to practical implementation [1,2]. Global integrated pest management (IPM) frameworks have further elevated the demand for timely, distributed monitoring data [2].

Capacitive sensing is a recent approach to automated insect detection. The technique is non-contact, low-power, and responsive to the dielectric properties of biological targets. Because insects have a body water content of approximately 60–80%, their passage through a capacitive sensing field induces a measurable change in capacitance [3,4,5,6,7,8,9,10,11,12]. The difference in permittivity between insect tissue and air is the basis of the detectable signal.

### 1.2. Physical Basis of Capacitive Sensing for Insect Detection

#### 1.2.1. Insects as Dielectric Bodies: A Biophysical Model

In electrical terms, an insect body can be modeled as a multilayered dielectric structure. The exoskeletal cuticle has a relative permittivity of approximately ε_r_ ≈ 5–7 and resistivity on the order of 10^6^–10^8^ Ω·m, serving as an outer insulating layer [6]. Hemolymph, comprising roughly 60–80% of body mass, has ε_r_ ≈ 70–80 at 1 kHz because of its ionic content [9,11]. Muscle tissue (ε_r_ ≈ 55–65) and body fat (ε_r_ ≈ 4–6) form additional dielectric layers. This stratified structure makes the insect body analogous to a lossy capacitor whose equivalent impedance varies with excitation frequency, moisture content, and temperature. Inter-species body size differences are substantial, with up to a 25-fold variation in body cross-sectional area observed across the six species examined in this study; however, the direct relationship between body morphology and dielectric sensing performance requires further quantitative validation.

When an insect enters the capacitive sensing channel, it partially displaces air (ε_r_ = 1) between the electrodes, thereby increasing the effective permittivity of the sensing gap. Extending the parallel-plate model, the capacitance change can be approximately expressed as:

(1) $$∆C\propto V_{insect}\times \frac{\varepsilon_{insect}-\varepsilon_{air}}{d^{2}}$$

where *V*_insect_ is the effective volume of the insect within the sensing region, ε_insect_ is its bulk equivalent permittivity, and d is the electrode spacing. That is, ΔC is jointly driven by body volume and permittivity contrast—larger insects produce stronger signals, and the high-water content of insects (ε >> 1) is the primary source of the capacitive signal.

Nelson [4] measured the dielectric properties of multiple insect species across radio-frequency and microwave bands, reporting that the dielectric loss factor increases with temperature (as ionic conductivity rises) and decreases with frequency. Ikediala et al. [9] reported that codling moth larval permittivity, and Wang et al. [10] confirmed that permittivity of other insect pests can exceed 40 at 27 MHz. Takikawa et al. [7] observed that dehydrated houseflies showed no response to electric fields, while hydrated specimens produced clear conductive pathways, supporting the hypothesis that body moisture is the primary source of the capacitive detection signal. Petrauskas et al. [12], in a review of organic electronics for insect sensing, noted that the high permittivity and body-water conductivity of insects make them suitable targets for electrical detection, but that research in this direction remains limited.

#### 1.2.2. Systematic Comparison of Sensing Modalities for Insect Detection

Automated insect detection can employ various sensing principles, each with distinct applicability and limitations. Capacitive sensing occupies a middle ground between low-information, low-cost binary sensors and high-information, high-cost modalities, as summarized in Table 1.

For dense, distributed field networks, capacitive sensing is a plausible low-power and low-cost option among the representative implementations summarized in Table 1. In addition, it remains insensitive to darkness, optical fouling, and alignment precision.

Optical camera systems [13,14,15] perform the best in species identification, but they consume substantially more power than capacitive sensing and require periodic lens cleaning. Optoacoustic sensors [16,17,18] can distinguish insect groups by wingbeat frequency, but perform poorly on wingless or low-frequency species and remain susceptible to ambient noise [18,19]. IR break-beam sensors [20,21] provide only presence/absence detection. Radar entomology [11,19] is effective for long-range migration monitoring but is expensive and technically demanding. Capacitive sensing therefore addresses a specific technical gap: it can provide more information than a binary gate sensor while remaining far more deployable than high-power imaging or radar systems.

### 1.3. Literature Review

The following review organizes existing work on automated insect detection by sensing principle, highlighting the specific limitations each method encounters in field deployment.

Among the earliest approaches, infrared counting systems use break-beam sensors to register insects entering traps. Jiang et al. [20] demonstrated a GSM-based wireless system for remote monitoring of *Bactrocera dorsalis*. Practical deployment, however, exposes environmental challenges: wind induces false triggers, strong sunlight saturates sensors, fog and dew scatter the optical path, and long-term use degrades optical elements [13,21,22]. To compensate for these limitations, Molina-Rotger et al. [23] added computer vision and machine learning for fruit fly identification, and Van-Khanh et al. [24] proposed an electronic trap design with long-term feature data acquisition for automated counting.

Optoacoustic and wingbeat sensing extracts species information from wingbeat frequency signatures. In a representative fruit-fly monitoring study, Potamitis et al. [16,17,18] reported a best classifier accuracy of 91.63% for *B. oleae* in an optoelectronic/optoacoustic trap system; related work by the same group also advanced automated surveillance hardware and bimodal optical sensing platforms [17,18]. Sandrini Moraes et al. [25] developed optoelectronic sensors for discriminating *Anastrepha fraterculus* and *Ceratitis capitata*; Kalfas et al. [26] applied convolutional neural networks (CNNs) to identify wingbeat features. These methods, however, remain sensitive to ambient light and wind-induced noise [21], and optical elements require periodic cleaning. In a related but technically distinct direction, Odgaard et al. [27] combined atmospheric electric field measurements with machine learning (precision ≈ 0.77, recall ≈ 0.81), sensing far-field perturbations rather than the near-field fringe capacitance exploited in the present study.

Camera-based methods using machine learning-based detection [13,14,15] can achieve species identification accuracies above 90% under favorable lighting, but per-node costs typically reach several hundred USD [13,14] and power consumption ranges from 0.5–4 W, limiting deployment in power- and budget-constrained field environments. Chen et al. [28] explored low-cost sensors combined with machine learning classifiers; Gardiner et al. [29] explored ultra-lightweight CNNs as on-device triggers to reduce computational load. Yet neither effort resolved the cost–power trade-off for large-scale field networks.

Work on capacitive sensing for insects is more recent and considerably sparser. Scherer et al. [3] used PCB capacitive pads to detect cockroach passage, measuring capacitance shifts of 5.71–15.3 fF. Campbell et al. [5] developed a capacitive bridge sensor for bee passage monitoring, combining capacitance changes with time-series analysis to track colony activity. Brendan Khoo T. et al. [30] designed a capacitive mosquito wingbeat sensor as a detection module for intelligent traps. van Klink et al. [31] conducted a comprehensive review of emerging sensor technologies for insect ecology, including optical, acoustic, radar, and capacitive modalities, and identified the lack of standardization and limited environmental robustness as critical unresolved challenges. Montgomery et al. [32] subsequently proposed standards and best practices for insect monitoring and benchmarking, emphasizing the need for reproducible protocols across sensing modalities. Despite these broader frameworks, no prior study has systematically examined electrode configuration, sensor IC characteristics, and target species body size together. While machine learning has been applied to optical and wingbeat-based sensing pipelines [26,29], its application to capacitive waveform classification remains largely unexplored. The present benchmarking dataset may therefore provide a useful foundation for future feature-extraction and species-discrimination studies.

This study focuses on establishing a controlled laboratory benchmark for capacitive sensing of adult terrestrial arthropods under a fixed dual-arc geometry (6 mm ID, 25 ± 1 °C, tethered transit), providing configuration-specific architecture-comparison and body-size-scaling reference that constitute a starting point for, rather than a substitute for, subsequent geometric, taxonomic, thermal, and field-deployment studies.

### 1.4. Capacitive Sensor IC Technologies

Commercial CDC ICs reflect different design philosophies and applications. The four devices examined here span precision Σ–Δ CDCs (FDC1004, AD7746) [33,34] and touch-oriented charge-transfer ICs (CAP1298, MPR121) [35,36], which differ in output mode, channel count, sampling behavior, and internal architecture, and are therefore expected to perform differently in insect detection.

Rather than a side-by-side comparison, this study organizes its single-configuration results along three analytic axes: geometry-driven signals, architecture-driven noise, and application-driven device selection. Within the tested 6 mm dual-arc conduit and six adult terrestrial species, it delivers configuration-specific response models, body-size and architecture effects on usable SNR, frozen-to-live conversion factors, and geometry-specific rules for CDC selection and conduit sizing. Extension to other electrode topologies, taxa, and free-flight conditions constitutes future work (Section 4.5).

## 2. Materials and Methods

### 2.1. Fundamentals of Capacitive Sensing

Capacitive sensing is based on the parallel-plate capacitor model:

(2) $$C=\frac{\varepsilon A}{d}$$

where ε denotes the permittivity of the dielectric medium, A represents the electrode area, and d is the separation distance between electrodes.

In practical insect detection, the sensing geometry is open rather than fully enclosed; so, the fringing field dominates. Sensitivity therefore depends not only on the dielectric properties and volume of the target, but also on its trajectory through the sensing field.

To avoid treating the tested devices as a simple product ranking, Table 2 summarizes them only at the architecture level. The purpose of this comparison is to define the main design axes relevant to capacitive insect sensing—absolute versus relative output, architecture-dependent noise behavior, and temporal capability—because these factors determine how each class should be interpreted in later analyses of static response, transient waveform capture, and humidity robustness.

The four ICs can be categorized into two architectural groups:

- Absolute-measurement ICs (FDC1004 [33], AD7746 [34]). These devices output calibrated capacitance values in physical units (pF/fF), enabling direct quantitative analysis of capacitance perturbations.
- Relative-change ICs (CAP1298 [35], MPR121 [36]). These devices report baseline-relative changes or threshold-crossing events and are primarily designed for touch or proximity sensing.

A key question for this study is whether the apparent laboratory advantage of ultra-high nominal resolution persists once field noise, environmental variation, and transit dynamics are considered.

### 2.2. Theoretical Basis and Experimental Design Strategy

To establish a generalizable and scientifically grounded design methodology for capacitive insect detection systems, this study integrates dielectric theory with controlled experimental validation. The sensing mechanism is governed by dielectric perturbation, indicated by Equation (1). This formulation indicates that capacitance response is jointly determined by (i) insect morphology and dielectric properties, (ii) electrode geometry, and (iii) sensor electronics. Therefore, a three-factor decoupling experimental strategy was adopted.

### 2.3. Experimental Setup

All experiments were conducted in an environmental chamber at 25 ± 1 °C, with relative humidity adjustable from 40% to 80% RH. Temperature was maintained at 25 ± 1 °C throughout all experiments and was not treated as an experimental variable. Consequently, its effects on body-fluid conductivity, electrode dielectric properties, and baseline drift remain uncharacterized in the present dataset. A custom nRF52840-based [37] acquisition board communicated with each CDC IC over I^2^C at 400 kHz.

The shared sensing conduit consisted of an acrylic tube (OD, 8 mm; ID, 6 mm; wall thickness, 1.0 mm; dielectric constant, 3.2) with two external copper arc electrodes (arc angle, 158.5°; axial width, W = 8 mm; circumferential length, 11.07 mm) separated by two 1.5 mm gaps. This electrode–IC combination served as the baseline configuration for subsequent field-relevant SNR estimate (SNR_FR) analysis.

The electrode configuration was selected based on a systematic comparison of two tube sizes and four electrode widths (Table 3). Among the 8 configurations tested, acrylic OD8/ID6 with W = 8 mm achieved an optimal balance between baseline capacitance and saturation avoidance; further widening to 10 mm provided only a negligible improvement (~0.5%).

**Figure 1:**
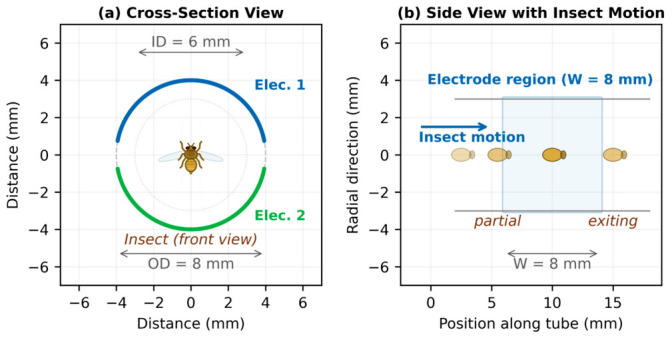
Schematic diagram of the experimental setup: (**a**) Cross-Section View (**b**) Side View.

Capacitance data were sampled at the configured rates used in this study (FDC1004: 400 Hz; AD7746: 90 Hz; CAP1298: 140 Hz; MPR121: 256 Hz) and logged to a microSD card via Serial Peripheral Interface (SPI). A custom Python 3.14.3 script running on a host PC performed real-time data visualization and post-processing. Figure 1 shows a schematic diagram of the experimental apparatus.

### 2.4. Phase 1: Dielectric Modeling

Three sponge sizes were used to simulate targets with controlled water content:

- Sponge A (small): Dimensions: 5 × 5 × 5 = 125 mm^3^ = 0.125 cm^3^.
- Sponge B (medium): Dimensions: 5 × 5 × 10 = 250 mm^3^ = 0.250 cm^3^.
- Sponge C (large): Dimensions: 5 × 5 × 15 = 375 mm^3^ = 0.375 cm^3^.

The balanced factorial design included:

- Water content: 40%, 45%, 50%, 55%, 60%, 65%, 70% (7 levels).
- Ambient humidity: 40%, 50%, 60%, 70%, 80% RH (5 levels).
- Sensing ICs: FDC1004, AD7746, CAP1298, MPR121.

For each condition, a sponge was hydrated to the target water content using deionized water, equilibrated in the chamber, inserted into the sensing region, and measured for 150 consecutive samples after signal stabilization. The Phase 1 analysis included capacitance–water-content regression, sensitivity, coefficient of variation (CV), volume-effect comparison, and humidity-response stability.

### 2.5. Phase 2: Biological Validation

#### 2.5.1. Test Species

Table 4 summarizes six species (0.79–19.63 mm^2^) that were used to test under frozen and live conditions to validate scaling behavior and assess biological variability. In addition to the five insect species, a spider was included as a representative non-target organism, as such organisms frequently interfere with detection in field traps. Its capacitance response was measured to assess potential interference and to explore preliminary methods for distinguishing non-target signals in practical deployment.

The present biological dataset is intentionally limited to adult terrestrial arthropods relevant to orchard and farmhouse-adjacent monitoring traps. Larvae, semi-aquatic insects, and soft-bodied taxa differ in dielectric properties and locomotion kinematics and therefore fall outside the scope of the current deployment.

A simplified signal-to-noise-ratio-based detection criterion is provided in Supplementary Materials File S1.

#### 2.5.2. Measurement Methods

Two measurement states were evaluated: frozen and live. For frozen specimens, individuals (within 24 h post-mortem) were attached to a nylon filament and pulled through the conduit using a stepper-motor-driven linear stage at transit speeds of 10, 20, and 30 mm/s. Tether-plus-stepper-motor actuation was selected to control transit speed, axial position, and body orientation within ±5% across all 720 condition cells. This isolates IC architecture as the primary variable and characterizes the deterministic, geometry-driven signal envelope. For live insects, a filament was bonded to the body surface, and specimens were driven through the sensing conduit using the same stage and identical speed conditions.

Phase 2 experimental design

A full factorial design was implemented with the following factors:

- Species (6 levels).
- Measurement state (frozen, live).
- Transit speed (10, 20, 30 mm/s).
- Relative humidity (40–80% RH; 5 levels).
- Sensing IC (4 types).
- This yielded a total of 720 experimental combinations.

For each IC × species × state × speed × humidity condition, 50 pass-through trials were planned. For each species, we recorded the number of individual specimens used, the median number of trials contributed per specimen, and the specimen-replacement criterion. To minimize fatigue in live measurements and dehydration in frozen measurements, specimens were rotated across trials and replaced according to predefined limits.

A replicate was defined as a single stage-controlled pass-through trial under a fixed IC × species × state × speed × humidity condition, with 50 trials planned per condition. A trial was considered valid only if it showed a stable pre-event baseline, uninterrupted passage through the sensing region, and an artifact-free waveform. Data quality was described by n_valid, waveform-retention ratio (n_valid/50), event-yield rate (n_detected/50), and, where relevant, conditional detection rate among valid waveforms (n_detected/n_valid).

#### 2.5.3. Analysis Methods

Phase 2 analysis was performed at both the trial and condition levels. Trial-level analysis included baseline characterization, event qualification, and waveform extraction, while condition-level analysis summarized mean response, SD, SNR, detection rate, frozen-versus-live comparison, inter-species discriminability, body-size correlation, and overall inter-IC performance under shared geometry and humidity conditions.

### 2.6. Signal Processing and Metrics

#### 2.6.1. Signal Features

Capacitive responses were analyzed using both static and dynamic features:

ΔC_static: steady-state dielectric loading while the insect occupies the sensing region.
ΔC_transit: transient waveform amplitude during passage through the sensing region.

Peak amplitude was selected as the primary transit feature because peak SNR directly quantifies the maximum signal margin, which is a prerequisite for reliable extraction of higher-order morphological features (e.g., rise time, fall time, FWHM, and multimodal structure) in future classification or species-discrimination tasks.

Signal quality was evaluated using distinct metrics for different analytical purposes. SNR_lab denotes the mean signal divided by intrinsic IC noise (σ_IC). SNR_FR denotes the deployment-oriented signal-to-noise ratio used in Section 4.3. SNR_IQR denotes an interquartile-range-based robustness metric for thresholded event detection. When a generic composite-noise illustration is needed, α = β = 0.5 is used unless otherwise stated; however, the deployment-oriented SNR_FR reported later should not be interpreted as identical to the generic composite metric in Equation (3).

(3) $$SNR=\frac{\alpha \cdot \Delta \text{C\_static}+\beta \cdot \Delta \text{C\_transit}}{\sqrt{{\left(\text{σ\_IC}\right)}^{2}+{\left(\text{σ\_lab}\right)}^{2}}}$$

#### 2.6.2. Thresholding, Event Count, and Valid-Sample Definition

Event qualification was based on an IQR-based threshold derived from the baseline distribution for each condition. A replicate was counted as a detected event only when the transient excursion exceeded the corresponding threshold and the waveform satisfied the valid-replicate criteria described above. Accordingly:

- Planned replicates = attempted pass-through trials specified by the design.
- Valid replicates = trials retained after waveform-quality screening.
- Events detected = valid replicates with threshold-positive transient responses.
- Data points = total waveform samples contributing to the corresponding event summary.

#### 2.6.3. Sampling Adequacy

Sampling adequacy was evaluated alongside detection thresholds because accurate transient reconstruction requires more than Nyquist-compliant event detection. Beyond meeting the minimum sampling-rate requirement, reliable extraction of peak amplitude, slope, and FWHM depend on sufficient sampling density across each pulse; accordingly, sampling adequacy was classified as Excellent (≥100 points), Good (50–99), Acceptable (20–49), or Insufficient (<20). To keep the inference process explicit, the statistical workflow was structured into model fitting, comparison, and validation steps, using regression for capacitance–water-content and body-size–response relationships, standard deviation and coefficient of variation for repeatability, paired tests or Wilcoxon signed-rank tests for frozen-versus-live comparisons, and event-level SNR, detection rate, humidity robustness, and sampling adequacy for performance evaluation.

## 3. Results

### 3.1. Static Sponge Model Results

#### 3.1.1. Role of Sponge Experiments

The sponge experiments (Phase 1) were not designed to “simulate insects,” but to calibrate the sensing system itself. Using standardized samples with known cross-sectional area (25 mm^2^) and controllable water content (40–70%), four key metrics were validated in Table 5.

The sponge experiments confirmed that the “ruler” itself is accurate—only then does measuring insects with it become meaningful.

#### 3.1.2. Linearity and Sensitivity Comparison

Under the baseline RH 40% condition, all four ICs showed highly linear capacitance–water-content relationships (R^2^ = 0.9878–0.9970) with similar sensitivities (6.17–6.56 fF/%), indicating that the static response slope was governed mainly by the shared electrode geometry rather than by IC architecture. However, repeatability differed markedly across architectures: the precision Σ–Δ CDCs showed much lower CVs (FDC1004: 0.98%; AD7746: 1.21%) than the charge-transfer ICs (CAP1298: 6.61%; MPR121: 4.34%), demonstrating superior short-term stability under controlled dielectric loading (Table 6).

#### 3.1.3. Volume Effect Analysis

**Figure 2:**
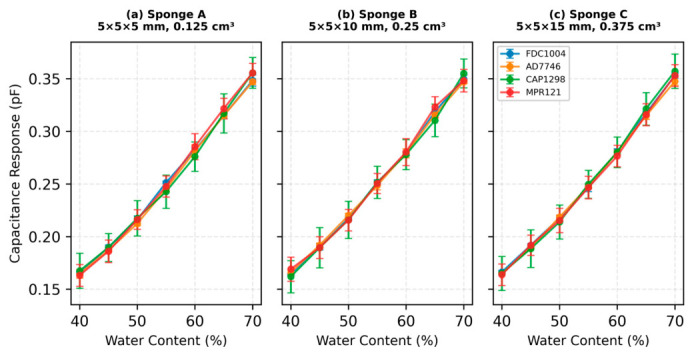
Capacitance response curves of the four ICs.

Under identical conditions, all three sponge volumes showed highly linear responses, with R^2^ values of 0.9878–0.9970 across all four ICs. Sensitivity variation over 0.125–0.375 cm^3^ remained below 5% (e.g., FDC1004: 6.14–6.24 fF/%), indicating that calibration was driven mainly by water content, with only a minor effect from surrogate-volume variation. The corresponding response curves are shown in Figure 2.

#### 3.1.4. Dielectric Differences: Sponge vs. Insect

**Figure 3:**
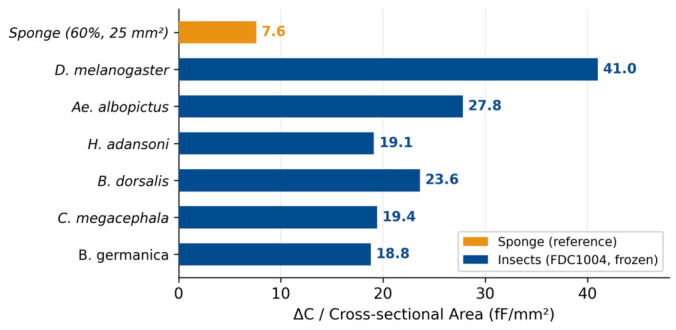
Comparison of ΔC per cross-sectional area (fF/mm^2^). Orange = sponge (porous matrix); blue = insects (FDC1004, continuous hemolymph). The dielectric efficiency difference is substantial.

At comparable water content (58–66%), insects exhibit 2.5–5.4× higher ΔC per unit cross-sectional area than sponges (Figure 3).

The reason: Water in sponges is dispersed within an open porous matrix (εr_eff ≈ 7.9), whereas insect hemolymph is a concentrated, continuous liquid phase (εr ≈ 70–80). The effective permittivity is far higher than that of porous materials at the same water content.

#### 3.1.5. Relationship Between Two Regression Lines

**Figure 4:**
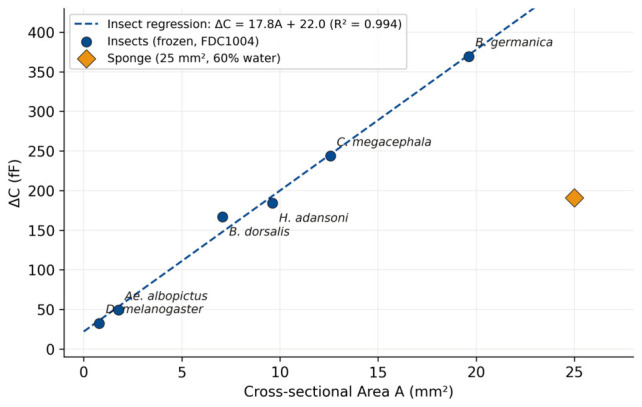
ΔC vs. cross-sectional area. Blue circles = insects (frozen, static measurement); orange diamond = sponge (60% water). Dashed line = insect regression.

The ratio between the sponge slope of 7.6 fF/mm^2^ (at 60% water) and the insect slope of 17.8 fF/mm^2^ (≈2.3×) reflects the effective dielectric multiplier between biological tissue and porous materials. This ratio stabilizes at 2.5× for larger insects (≥7 mm^2^) and rises to 5.4× for smaller insects, likely due to electric field concentration effects at the tube center (Figure 4).

The value of sponge calibration lies not in predicting the absolute ΔC of insects, but in:

(1) Validating the sensing system’s linearity, sensitivity consistency, and repeatability establishing measurement tool credibility;
(2) Providing a reference anchor with known dielectric properties, making insect measurements traceable to a physical model;
(3) Separating “geometric factors” (cross-sectional area) from “material factors” (permittivity), providing a physical basis for the ΔC = kA + C_0_ regression.

### 3.2. Insect Detection Results

#### 3.2.1. FDC1004 Insect Pass-Through Measurement Summary

Table 7 presents the FDC1004 pass-through measurement statistics for all six insect species under standard conditions (RH 40%, 10 mm/s, frozen specimens). FDC1004 is presented first because its 400 SPS sampling rate, the highest among the four ICs, provides the most complete waveform dataset and serves as the baseline reference for subsequent comparisons with the other ICs (Section 4.3). Each row reports the baseline capacitance statistics of the empty channel, the mean and standard deviation of transit capacitance change (ΔC_transit) and event-level SNR, the number of detected events (n_detected), the event-yield rate (n_detected/50), and its 95% confidence interval.

**Figure 5:**
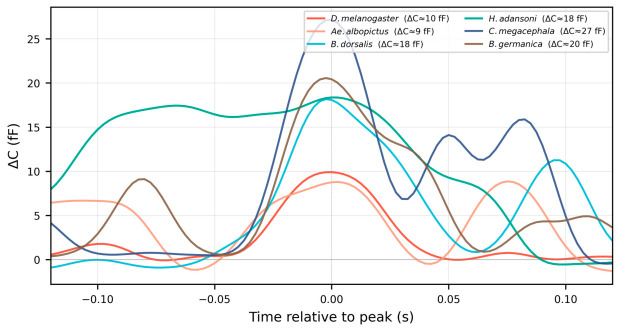
Peak-aligned FDC1004 capacitance response (ΔC) for six insect species during single pass-through events.

Figure 5 shows peak-aligned FDC1004 transit waveforms (Δ*C* relative to baseline) for the six species. Despite being the largest species, *B. germanica* produced a lower mean transit Δ*C* (16.72 ± 5.96 fF) than *B. dorsalis* (18.15 ± 4.38 fF) or *H. adansoni* (18.26 ± 1.98 fF), because transit Δ*C* depends on posture, speed, and gap-occupancy fraction rather than body volume alone (cf. the strict ΔC_static–area linearity in Section 3.3.1). Conversely, *B. germanica* exhibited the highest event-level SNR (6.08 ± 2.17), whereas *D. melanogaster* yielded the lowest Δ*C* (8.56 ± 2.89 fF) but still maintained an event-level SNR of 5.44 ± 1.84 because of its brief but sharp peak. Inter-species variability also differs: *Ae. albopictus* shows the tightest Δ*C* scatter (SD = 0.54 fF) while *C. megacephala* the widest (SD = 7.50 fF), reflecting postural variation during passage. These differences suggest that threshold selection may need to be species- or scenario-specific in future implementations. For *Ae. albopictus* (*n_detected* = 5, event-yield rate 10%) and *D. melanogaster* (*n* = 7, 14%), the low event counts should be interpreted as an operational indicator of detection feasibility rather than a sampling failure. Together with the SNR_FR analysis in Section 3.3.1, these results provide convergent evidence that the current 6 mm ID geometry is near or below the practical detection limit for body cross-sections below approximately 2 mm^2^.

#### 3.2.2. Dynamic Event Amplitude Ranking by Species

Dynamic transit events represent rapid waveform changes during passage and are quantified as peak-to-baseline amplitudes, distinct from static ΔC_static (Section 3.3.1). Dynamic amplitudes showed substantial overlap across species and did not follow a monotonic body-size trend, reflecting posture- and motion-dependent variability.

#### 3.2.3. Baseline Stability for Dynamic Event Detection

Baseline stability was evaluated using the coefficient of variation (CV) of the empty-channel signal, because unstable baselines directly affect threshold-based event qualification. Table 8 classifies baseline stability according to CV and summarizes its practical implications for false positives, false negatives, and small-insect detectability.

**Figure 6:**
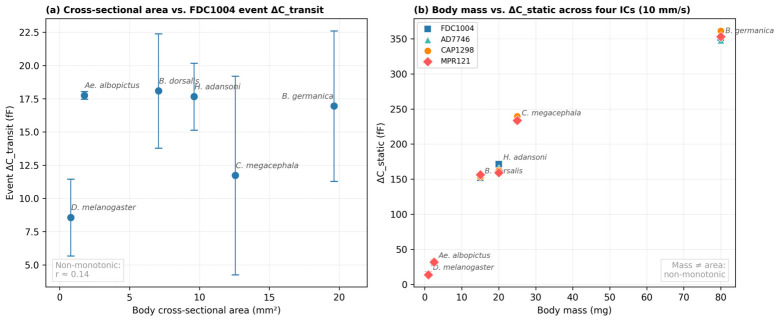
Non-monotonic relationship between insect body dimensions and dynamic event ΔC_transit: (**a**) body cross-sectional area vs. FDC1004 event ΔC_transit for six species; (**b**) body mass vs. event ΔC_transit across all four ICs at 10 mm/s. Note: Dynamic transit ΔC differs from static ΔC_static (Section 3.3.1), which exhibits strong linearity (r = 0.995).

Figure 6 illustrates the relationship between body size and dynamic transit ΔC. Unlike static ΔC_static, which scales linearly with cross-sectional area (17.96 fF/mm^2^, r = 0.995; Section 4.3), transit ΔC does not increase monotonically with body size—posture, body geometry, and water distribution during passage all modulate the transient signal amplitude. Although static measurements offer more reliable size estimation, practical field deployment relies on dynamic pass-through detection, making transit ΔC the operationally relevant metric.

### 3.3. Cross-IC System-Level Analysis

To support configuration guidance, system-level performance was analyzed along five dimensions: field-relevant SNR estimate, static sensitivity, detection limit, environmental stability, and sampling adequacy.

Three SNR metrics were used:

(1) SNR_lab: mean signal divided by intrinsic IC noise (σ_IC).
(2) SNR_FR: deployment-oriented SNR.
(3) SNR_IQR: an IQR-based robustness metric used for thresholded event detection.

The field-relevant SNR estimate was defined as SNR_FR:

(4) $$\text{SNR\_FR}=\frac{\Delta C_{static}\times \kappa }{\sqrt{{\text{σ\_IC}}^{2}+{\text{σ\_lab}}^{2}}}$$

where *κ* = 0.819 is the geometric fill factor of the dual-arc conduit, σ_IC is the intrinsic IC noise, and σ_lab = 5 fF is a scenario parameter adopted for deployment-oriented sensitivity analysis. This value should not be interpreted as a universally measured field constant; rather, it provides a first-order benchmark whose influence is examined explicitly in Section 4.3. The sensitivity of all conclusions to this parameter is also examined in Section 4.3.

#### 3.3.1. Field-Relevant SNR Estimate and Detection Limit

Three observations emerge from Table 9. First, ΔC_static was only weakly IC-dependent, and inter-IC differences for the same species remained modest. Second, the ranking of the field-relevant SNR estimate was consistent across species: AD7746 > FDC1004 >> MPR121 > CAP1298. Third, small insects remained challenging. *D. melanogaster* fell below the practical SNR_FR = 3 threshold for all four ICs, whereas *Ae. albopictus* remained below this threshold for the charge-transfer devices.

**Figure 7:**
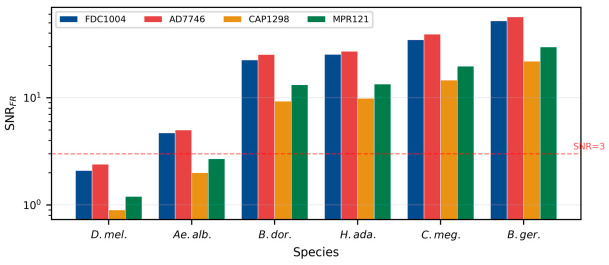
Cross-species SNR comparison of the four ICs.

Figure 7 demonstrates that the observed differences primarily arise from the intrinsic characteristics of the ICs, rather than incidental advantages associated with specific species. As body size increases, SNR_FR rises and inter-IC differences widen: *D. melanogaster* values cluster tightly (2.1/2.4/0.9/1.2), whereas *B. germanica* diverges markedly (52.1/56.7/21.9/29.7), because larger ΔC_static amplifies the effect of differing noise floors. For the smallest species (*D. melanogaster*, *Ae. albopictus*), all ICs hover near or below SNR = 3, with only Σ–Δ devices reaching the detectable range—here, IC selection determines detection feasibility, not merely performance.

#### 3.3.2. Static Capacitance Scaling with Body Size

Static capacitance change, measured as the sustained DC offset while the insect occupied the sensing region, showed strong linearity with body cross-sectional area across all four ICs. The pooled regression was:

(5) $$\Delta \text{C\_static}=17.96\;fF/{mm}^{2}\times \text{A\_cross}+5.07\;fF$$

with *r* = 0.995 and *R^2^* = 0.990.

Across the four ICs, the sensitivity coefficient remained within 17.76–18.32 fF/mm^2^, and the offset term remained within 3.54–6.45 fF, indicating that static response was governed primarily by body-size occupancy within the sensing field under the tested geometry. Within the present six-species benchmark, ΔC_static therefore serves as a first-order proxy for body cross-sectional area.

Importantly, all ICs produced ΔC_static values well above their intrinsic noise floors; however, in the deployment-oriented comparison, *D. melanogaster* remained below the practical SNR_FR = 3 threshold. This suggests that reliable detection of insects with cross-sectional areas below approximately 2 mm^2^ would require further noise reduction, geometry optimization, or both.

#### 3.3.3. Stability and Reliability

**Figure 8:**
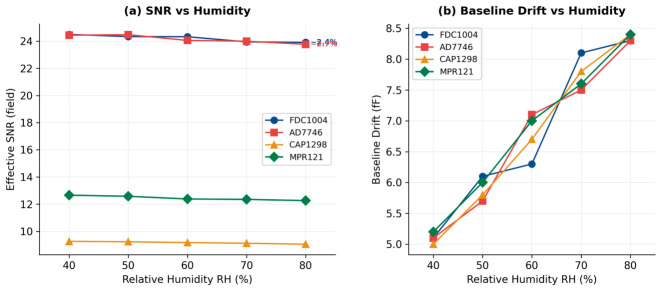
Humidity-dependent degradation of SNR and baseline drift across four capacitive sensing ICs (RH 40–80%, *N* = 50). (**a**) Effective field SNR. (**b**) Baseline drift.

Humidity-dependent degradation in sensing performance was evaluated for four capacitive ICs over RH 40–80% using an acrylic tube (OD 8 mm/ID 6 mm; N = 50 per condition). (a) Effective field SNR: The Σ–Δ ICs (FDC1004 and AD7746) maintained SNR_lab values above 23.7, with static sensitivity degradation of only 2.4% (<3.2%) across the full humidity range (RH 40–80%). Under the deployment-oriented composite noise model (SNR_FR), however, the same devices exhibited up to 35.5% degradation due to the amplified effect of humidity-induced baseline drift on the overall noise floor (see Section 4.3), whereas the charge-transfer ICs (CAP1298 and MPR121) plateaued at approximately 9–13. (b) Baseline drift increased monotonically from approximately 5 fF at RH 40% to approximately 8.4 fF at RH 80%, and all four ICs showed a similar trend, indicating that the drift was governed primarily by the electrode–tube interface rather than by IC architecture (Figure 8).

#### 3.3.4. Deployment Feasibility

**Figure 9:**
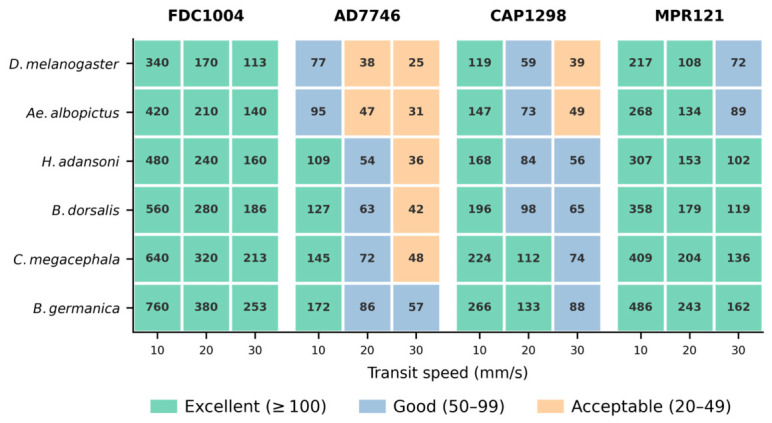
Sampling points per transit event for six insect species at three transit speeds (10, 20, 30 mm/s) across four ICs. Cell values indicate the number of sampling points; color coding reflects adequacy rating.

Figure 9 summarizes sampling-point counts and adequacy across all species–speed–IC combinations (*N* = 50 per condition). Sampling density varied nearly 30-fold, from 754 ± 9 points (FDC1004, *B. germanica*, 10 mm/s) to 25 ± 1 points (AD7746, *D. melanogaster*, 30 mm/s), mainly due to body size and transit speed, which together determine the effective sampling window.

Among the four ICs, FDC1004 (400 SPS) consistently provided excellent waveform reconstruction, while MPR121 (256 SPS) also performed well with only minor loss under the most demanding conditions. CAP1298 (140 SPS) showed intermediate performance, whereas AD7746 (90 SPS) was the main limitation for high-speed detection of small insects. Overall, sampling rate was the dominant constraint, with at least 256 SPS needed for robust performance and 400 SPS providing the most reliable reconstruction. Sampling-point counts should therefore be interpreted relative to the effective event window rather than body length alone.

Although CAP1298 and MPR121 exhibit the lowest power consumption, their measurement precision limitations suggest suitability primarily for threshold-triggered binary detection rather than quantitative analysis.

### 3.4. Frozen Specimen vs. Live Insect Measurements

Research objective 3 focuses on quantifying the differences between frozen and live insect measurements in order to derive conversion factors for translating laboratory calibration to field deployment. This objective was addressed through paired statistical testing, variability comparison, and conversion-factor estimation.

Wilcoxon signed-rank tests were conducted on paired conditions defined by species, IC, RH, and transit speed, yielding 360 paired conditions in the full factorial design (6 species × 4 ICs × 5 RH levels × 3 speed levels). Bonferroni correction was applied for per-species comparisons (adjusted *α* = 0.0083).

Live measurements showed a slightly higher capacitance than frozen specimens (+0.75% ± 0.82%), although the absolute difference remained small (<1%). This indicates that calibration based on frozen specimens can be directly applied with minimal correction. Table 10 summarizes species-level CV values averaged across IC and RH after first averaging over the three transit speeds.

The difference in coefficient of variation (CV) between frozen and live measurements was species-dependent but generally small (<1.6 percentage points), indicating similar measurement stability under both conditions. Across all specimen conditions, the precision CDCs (FDC1004 and AD7746) consistently showed lower CV than the charge-transfer ICs, confirming superior repeatability.

Paired analysis further showed that frozen-to-live conversion factors (*k* = *C_live*/*C_frozen*) remained close to unity. For small species (<6 mm), *k* was approximately 1.00, indicating that frozen calibration can be used directly, while larger species showed a only minor deviation (*k* = 1.006–1.018). Overall, the mean capacitance difference between frozen and live specimens remained within 2%, supporting frozen-specimen calibration as a reliable baseline for field deployment with minimal correction (Table 11).

For the smallest species examined in this study, frozen calibration values can generally be used without correction. For larger species, modest species-specific correction factors may improve agreement between frozen and live measurements, typically on the order of 1.006–1.018 in the present dataset. Waveform-shape differences between frozen and live specimens, including dynamic components arising from limb oscillation and hemolymph pulsation, are beyond the scope of this amplitude-focused calibration comparison and represent a distinct analytical problem relevant to live/dead discrimination and debris rejection.

## 4. Discussion

### 4.1. Deployment-Oriented Interpretation and Scenario-Based Recommendations

**Figure 10:**
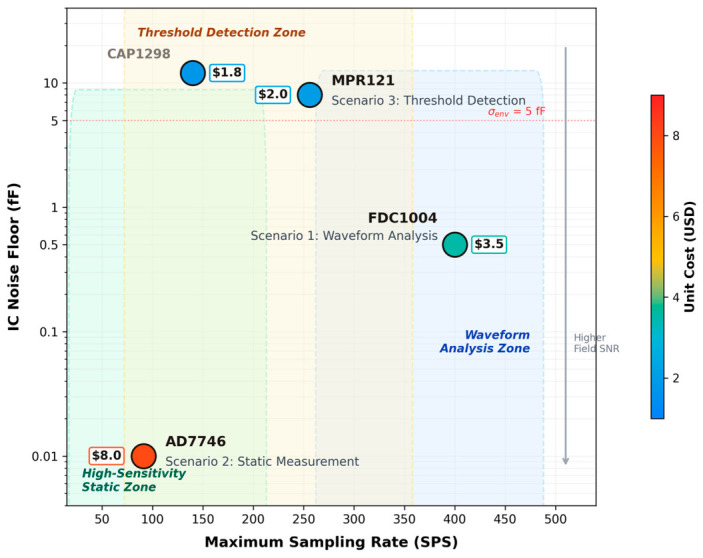
Decision map for CDC-based insect sensing system design.

The performance required of an electronic insect trap depends on the biological target, the lure–trap combination, and the role assigned to the sensing gate [1,2,32]. In practice, the trap system should be made as taxonomically selective as possible before signal processing is asked to discriminate events. Lure chemistry, trap geometry, and conduit design can all reduce bycatch, thereby lowering the burden placed on the sensing electronics. This interpretation should be supported primarily by fruit-fly trapping and management references rather than by sensor-benchmarking papers alone. For that reason, the present results are interpreted below in scenario form: the preferred CDC architecture depends not on a universal device ranking, but on whether the application prioritizes dynamic waveform capture, static occupancy measurement, or low-cost threshold triggering. Figure 10 summarizes this decision space.

Scenario 1: Configuration best suited to high-reliability dynamic monitoring under the present laboratory constraints (Recommended: FDC1004 + dual-arc electrode conduit). Within this benchmark, FDC1004 provided the most balanced solution for dynamic transit sensing. Its 400 SPS sampling rate supported robust waveform reconstruction during rapid pass-through events, its short-term variability remained low (CV = 0.55%), and its four-channel architecture offers a practical compromise among temporal resolution, noise, cost, and scalability. Under the deployment-oriented noise assumptions used in this paper, FDC1004 does not fully resolve the smallest insects, but it remains suitable for most target pests at or above the *Ae. Albopictus* size range. The conclusion should therefore be read as conditional: this configuration is the strongest first-pass option for dynamic waveform capture and conduit-level feasibility screening within the tested operating envelope, not the universal best device for every future field setting.

Scenario 2: Configuration best suited to high-sensitivity static or quasi-static measurements under controlled or semi-controlled conditions (AD7746 + dual-arc electrode conduit). AD7746 exhibited the lowest intrinsic noise floor among the tested devices and therefore the highest laboratory-limited SNR. Under the composite-noise assumptions used for deployment-oriented interpretation, however, its practical advantage over FDC1004 narrowed because total noise became increasingly dominated by environmental contributions rather than by front-end resolution alone. For example, under the baseline geometry at RH 40%, SNR_FR for *B. germanica* reached 56.7, but this estimate decreased by 35.5% at RH 80% in the humidity-dependent model.

AD7746 is therefore best regarded as a strong option for high-sensitivity static or low-speed measurements in controlled or semi-controlled environments, especially where humidity robustness matters. Its main practical limitations are the lower sampling rate (90 SPS), which constrains fast transit analysis, the two-channel architecture, which limits scalability, and the higher unit cost.

Here, static or quasi-static measurement denotes sustained occupancy measurement, in which the target remains within—or repeatedly occupies—the sensing zone long enough for a stable offset to be estimated. Binary presence/absence detection is one limiting case of this mode, but not the only one. The same configuration may also support slow or dwell-based quantitative measurements, for example, when monitoring whether *B. germanica* individuals occupy a bait station over an extended interval. This interpretation is consistent with the capacitive tunnel monitoring reported by Campbell et al. [5] and the PCB-based insect sensing approach reported by Scherer et al. [3], and it should be distinguished from dynamic transit sensing aimed at resolving individual pass-through waveforms to extract features such as body length, velocity, or species-discriminative characteristics.

Scenario 3: Reference configuration for low-cost threshold-triggered presence detection (MPR121 + simplified electrode conduit). In this scenario, trap structure and lure selectivity provide most of the taxonomic filtering; so, electronics only need to register the presence of an object within the sensing field. Under that assumption, a low-cost threshold-triggered network becomes feasible.

CAP1298 and MPR121 may still be useful for threshold-based presence detection when taxonomic specificity is provided primarily by trap structure or lure selectivity. However, they are not suitable for quantitative capacitance analysis because of their higher noise floors, lower repeatability, and relative-output format. Under the humidity-dependent SNR_FR model, their degradation from RH 40% to 80% reached 64.8% and 66.8%, respectively (Figure 8), indicating substantially greater sensitivity to combined intrinsic and laboratory baseline noise than the Σ–Δ devices. Their practical role is therefore limited to low-cost binary detection rather than calibrated waveform-based sensing.

The common intuition that a smaller percentage drop automatically implies better stability is misleading, because charge-transfer devices start from much lower baseline SNR values and remain more sensitive to substrate and air-gap dielectric variation. In addition, these devices report relative rather than fully calibrated capacitance changes, which restrict direct inter-device calibration and comparison.

Even so, where only binary triggering is required and cost or channel density dominates the design brief, the MPR121 12-channel format may still be attractive for large-scale threshold networks. The present experiments were performed under controlled laboratory conditions using a single electrode geometry. Translation to field operation will require additional verification of geometry transferability, environmental-noise characterization, fouling tolerance, and long-term baseline drift. As noted by van Klink et al. [31], environmental robustness and long-term drift remain major barriers for automated insect sensing technologies. The present results suggest likewise that field performance is likely to be limited less by nominal front-end resolution than by environmental electromagnetic and dielectric variability. Accordingly, the next design priority is not only component optimization, but also system-level noise mitigation, fouling control, and recalibration strategy.

The most critical unresolved issue is long-term stability under outdoor exposure, including UV, rain, dust, and temperature cycling. A logical next step is a season-long orchard pilot focused on baseline drift, fouling, and recalibration interval rather than assuming that laboratory stability will transfer directly to field conditions.

In parallel, multi-electrode conduits could extend the present single-channel design toward trajectory and velocity reconstruction without external speed control, building on prior capacitive sensing concepts and related multi-electrode implementations where directly relevant. This would expand the present framework from single-gate detection toward richer event characterization.

A separate deployment pathway is integration with baited trap architectures, in which methyl eugenol or cue–lure systems channel target fruit flies through the sensing conduit [38,39,40]. Such integration could improve encounter rate and trap-level inference, but it still requires dedicated mechanical design and field validation. Because node power consumption remains a major determinant of agricultural sensor-network lifetime [41], the low-power nature of capacitive sensing remains relevant at the system level.

### 4.2. First-Order, Geometry-Specific Design Reference for the Tested 6 mm Dual-Arc Conduit

The present findings were consolidated into a first-order design relation specific to the 6 mm dual-arc conduit, combining dielectric theory with an empirically fitted scaling coefficient. The relation is geometry-bounded: both the scaling coefficient *k* and the fill factor *κ* reported in this paper apply only to this configuration and must be re-derived for alternative electrode topologies or conduit diameters. Based on the capacitive perturbation mechanism, the detectable capacitance change can be approximated as:

(6) $$\Delta C=k\cdot A_{cross}+C_{0}$$

where *A*_cross_ represents the insect body cross-sectional area, *k* is the system sensitivity coefficient (fF/mm^2^), and C_0_ is the baseline offset determined by electrode geometry and parasitic capacitance.

From pooled experimental data, the scaling coefficient was obtained as:

(7) $$k=17.96\;fF/{mm}^{2}$$

For the baseline dual-arc conduit (acrylic OD 8 mm/ID 6 mm, W = 8 mm, arc angle 158.5°), the pooled scaling coefficient was *k* = 17.96 fF/mm^2^. This coefficient is geometry-specific and is expected to vary with conduit diameter, electrode coverage angle, and wall material through changes in fringe-field distribution and geometric fill factor *κ*. The strong linearity observed here (*r* = 0.995) indicates that static capacitance response is governed primarily by geometric occupancy within the sensing field. However, validation across alternative conduit geometries is required before *k* can be treated as a transferable design constant.

The practical detectability of a target species can be approximated using the field-relevant SNR estimate:

(8) $$\text{SNR\_FR}=\frac{\Delta C}{\sqrt{{\left(\text{σ\_IC}\right)}^{2}+{\left(\text{σ\_lab}\right)}^{2}}}$$

Note that Equation (8) omits the electrode-specific geometric fill factor *κ* (cf. Equation (4), where *κ* = 0.819 for the dual-arc conduit) and therefore provides an upper-bound estimate applicable to arbitrary electrode geometries. For a specific conduit geometry, the realized SNR may be lower by a factor of *κ*.

Here, σ_IC denotes intrinsic sensor noise and σ_lab denotes the assumed deployment noise floor. Accordingly, this formulation should be interpreted as a first-order design estimate rather than a directly measured field metric.

Within the tested geometry, this formulation provides an approximate, conduit-specific design reference that supports the following first-order uses:

- First-order screening of detection feasibility by body size, prior to physical prototyping.
- Preliminary CDC selection within the tested architecture set (sigma–delta vs. charge-transfer), based on SNR and measurement-range requirements.

Furthermore, the model indicates that system performance transitions from an IC-limited regime to an environment-limited regime when:

(9) $${\text{σ\_lab}}^{2}\gg {\text{σ\_IC}}^{2}$$

Under such conditions, improvements in ADC resolution or intrinsic IC noise yield diminishing returns, and overall performance becomes dominated by laboratory baseline noise sources. This relation is intended as a first-order, geometry-specific design reference for the 6 mm dual-arc conduit rather than a fully predictive physical model; its scaling coefficient and fill factor must be re-derived for any other electrode topologies, conduit diameters, life stages, or temperatures.

### 4.3. Sensitivity of Field-Relevant SNR to Deployment Noise Assumptions

As stated in Section 3.3.1, σ_lab is a reference value derived from laboratory measurements rather than a field-measured constant. The SNR_FR values obtained under this assumption should be interpreted as design estimates for preliminary CDC selection, not as direct predictors of field performance. The field-relevant SNR estimates presented in Section 4 rely on an assumed laboratory baseline noise floor σ_lab = 5 fF, representing a first-order estimate of deployment-site interference (electromagnetic interference, mechanical vibration, and dielectric fluctuations from airborne particulates). Because this parameter has not been measured in situ and will vary across deployment environments—ranging from electromagnetically quiet laboratory enclosures to high-EMI agricultural settings near irrigation pumps or power lines—it is important to assess the robustness of our IC ranking conclusions against this assumption.

**Figure 11:**
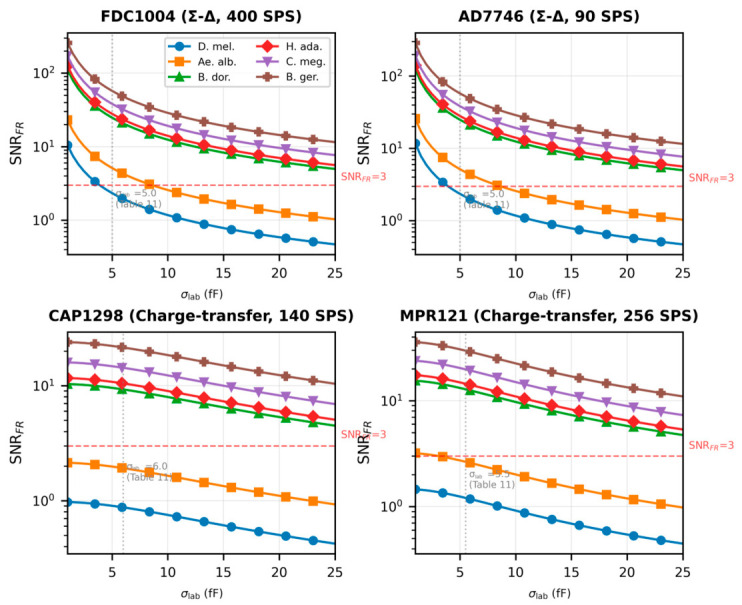
Sensitivity of field-relevant signal-to-noise ratio (SNR_FR) to the assumed laboratory baseline noise floor σ_lab (1–25 fF). The maximum tolerable σ_lab for each IC–species combination at SNR_FR = 3, and the stability of IC rankings as σ_lab increases. The dashed line indicates the baseline assumption of σ_lab = 5 fF. Across the tested range, the Σ–Δ devices consistently maintain higher SNR_FR values than the charge-transfer devices.

Figure 11 shows the theoretical sensitivity of SNR_FR to the assumed laboratory noise floor (σ_lab) for each CDC IC across six insect species. Curves were computed analytically as Equation (4), where ∆C_static is the species-specific static capacitance shift, *κ* = 0.819 is the geometric fill factor, and σ_IC is the intrinsic noise of each IC. The vertical dashed line marks the baseline assumption σ_lab = 5 fF used throughout the deployment-oriented analysis. This figure illustrates how the theoretical detection margin changes as laboratory baseline noise varies, independent of specific measurement replicates.

### 4.4. Sensitivity Analysis Revealed Three Key Findings

First, the IC ranking remained stable across realistic deployment conditions: For σ_lab > 1 fF, the Sigma-Delta ICs (AD7746 and FDC1004) consistently outperformed the charge-transfer ICs. The crossover between AD7746 and FDC1004 occurred only at very low σ_lab, below the range expected in practical deployment, indicating that AD7746 effectively retains the highest SNR_FR in the low-noise limit. Once laboratory baseline noise dominates (σ_lab ≫ σ_IC), inter-IC differences narrow and performance becomes governed primarily by ΔC_static and geometry.

Second, absolute sensitivity to σ_lab is strongly body-size dependent. Large species such as *B. germanica* remain detectable even under comparatively noisy conditions, whereas small species such as *D. melanogaster* approach or fall below the practical detection threshold near the baseline scenario considered here. This establishes a practical lower bound: detection of insects with a cross-sectional area below approximately 1 mm^2^ will require either lower laboratory baseline noise or a geometry that increases ΔC_static.

Third, the maximum tolerable laboratory baseline noise for maintaining SNR_FR ≥ 3 scales with body size, providing a practical deployment guideline. Site feasibility can therefore be assessed by measuring baseline capacitance variability in the target deployment environment, estimating the effective laboratory baseline noise term, and comparing that estimate against species-specific detectability thresholds.

Overall, although absolute SNR_FR values depend on the assumed σ_lab, the main conclusions—superior performance of the Σ–Δ architectures, stable architecture ranking, and body size as the primary determinant of detectability—remain robust across realistic deployment scenarios. Field deployment should therefore include site-specific environmental characterization. As a first-order estimate, published dielectric data [4] indicate that a 15 °C increase in ambient temperature raises hemolymph ionic conductivity by approximately 30–45%. Under the present geometry, this corresponds to a ΔC_static shift of roughly 3–5 fF for mid-sized species—comparable to the σ_IC floor of the FDC1004. However, baseline drift at the electrode–acrylic interface may be larger and is not bounded by this estimate.

### 4.5. Scope and Limitations

The findings apply within a deliberately bounded experimental framework; the principal limitations are listed below to avoid over-extrapolation.

(i) Electrode geometry. Only a single dual-arc geometry on a 6 mm ID acrylic conduit was tested (*κ* = 0.819, *k* = 17.96 fF/mm^2^). Parallel-plate, annular, interdigitated topologies, and 3–4 mm small-bore conduits were not examined; *k*, *κ*, and SNR_FR rankings must be re-derived for any other configuration.
(ii) Biological taxa. The six tested species were adult terrestrial arthropods. Larvae, semi-aquatic, and soft-bodied taxa lie outside scope. The ΔC_static–area linear relationship should not be extrapolated to them without dedicated calibration.
(iii) Temperature. The chamber was held at 25 ± 1 °C and not varied. Published dielectric data [4] suggest that a 15 °C ambient rise would yield a ΔC shift of < 5 fF for mid-sized insects under the present geometry, but interface-driven baseline drift is not bounded by this estimate.
(iv) Locomotion model. Specimens were translated by a tethered stepper-motor stage at 10 mm/s, suppressing wing-beat, body-torsion, and limb-oscillation components. The reported amplitudes therefore under-represent free-flight high-frequency content that would further favor the higher-sampling Σ-Δ architectures rather than reverse the present ranking.
(v) Statistical power for small species. Aedes albopictus (*n* = 5) and Drosophila melanogaster (*n* = 7) yielded valid detections well below the planned 50 per condition. These results are reported as feasibility screening, indicating that the present geometry approaches the detection limit near cross-sectional area ≈1–2 mm^2^, not as definitive statistical characterization.
(vi) Noise model. σ_lab = 5 fF was a literature-anchored scenario value, not an in situ field measurement; absolute SNR_FR values were design estimates, not field predictions. The architecture ranking, however, remained robust over σ_lab ∈ [1, 25] fF (Section 4.3, Figure 11).

Accordingly, this study is best read as a single-configuration laboratory benchmark and a conduit-specific design reference; extension beyond the tested framework is required in future studies.

## 5. Conclusions

This study established a single-configuration laboratory benchmark comparing four CDC ICs across six adult terrestrial species in a 6 mm dual-arc conduit. Static capacitance change scaled strongly with body cross-sectional area, supporting first-order feasibility screening, conduit sizing, and signal budgeting; the scaling coefficient must be re-fitted for alternative geometries (Section 4.5).

The results further show that sensing architecture was more consequential than nominal ADC resolution for environmentally robust measurement. The body-size scaling relationship and architecture ranking reported in this paper are valid within the scope of adult terrestrial arthropods in orchard and farmhouse-adjacent settings; generalization to other life stages or habitat types requires dedicated validation. Under the humidity-dependent noise model, Σ–Δ architectures retained substantially higher SNR_FR than charge-transfer devices across RH 40–80%, although both classes degraded measurably; for the Σ–Δ devices, the approximately 35.5% reduction was driven mainly by the assumed environmental-noise term. Static and dynamic measurements also served different purposes: static response supported size-related estimation and occupancy-style sensing, whereas dynamic transit response required waveform-based interpretation because posture and motion materially affected event shape.

Taken together, this study provides a single-configuration laboratory benchmark and a conduit-specific first-order design reference for the 6 mm dual-arc geometry tested. Before any field-general or cross-configuration claim, five priorities must be addressed: (i) site-specific characterization of the σ_lab = 5 fF baseline; (ii) long-term outdoor stability under UV light, rain, dust, and temperature cycling; (iii) trap-integrated validation with naturally occurring pest populations; (iv) controlled-temperature replication to bound thermal drift; and (v) free-locomotion trials and alternative electrode topologies (parallel-plate, annular, interdigitated, 3–4 mm small-bore) to test ranking and scaling outside the present configuration.

## Figures and Tables

**Table 1:** Multi-dimensional comparison of sensing technologies for insect detection.

Sensing Technology	Power	Cost per Node (USD)	All-Weather	Maintenance	Species Potential
Capacitive	Ultra-low (~1 mW active; 10–50 μW avg. with duty-cycling)	Low (USD 50–200)	Excellent (dark/rain/dust)	Very low	Moderate (multi-parameter)
Optical/Camera	High (>200 mW)	High (USD 300+)	Poor (needs light, fouling)	High (lens cleaning)	Excellent (visual features)
Optoacoustic/Wingbeat	Medium (30–100 μW)	Medium (USD 100)	Medium (wind/rain noise)	Medium	High (frequency features)
IR Break-beam	Medium (20–50 mW)	Low (USD 70)	Poor (rain/fog, alignment)	Medium	None (binary only)
Radar/mmWave	Very high (>500 mW)	Very high (USD 5000+)	Excellent	High (specialized)	Low (no database)

Values are representative literature- or datasheet-derived ranges compiled for deployment-oriented comparison; exact values depend on node architecture and operating mode.

**Table 2:** Architecture-level features relevant to capacitive insect sensing.

Parameter	FDC1004	AD7746	CAP1298	MPR121
Output units	Physical	Physical	Relative	Relative
Channels	4	2	8	12
ADC Resolution	16-bit	24-bit	8-bit (delta count)	10-bit
Measurement Range	±15 pF	±4 pF (expandable)	n.a.	n.a.
Capacitance Resolution	0.5 fF	4 aF	n.a. ^‡^	n.a. ^‡^
Sample Rate	100/200/400 S/s	10–90 Hz	140 Hz	1–256 Hz

^‡^ Touch-sensing ICs: fF values are engineering estimates from measured data.

**Table 3:** ΔC: FDC1004/AD7746 (pF); CAP1298/MPR121 (counts ^†^).

OD/ID (mm)	W (mm)	Area (mm^2^)	C_base (pF)	ΔC (pF)	FDC1004	AD7746	CAP1298	MPR121
8/6	4	88.5	0.0451	0.171	34	34	13.3	18.1
8/6	6	132.8	0.0674	0.256	51	51.2	19.6	27.1
8/6	8	177.1	0.0898	0.341	67.8	68.1	26.2	36.1
8/6	10	221.3	0.1119	0.342	67.9	68.4	26.3	36.3
10/8	4	113.7	0.0467	0.126	25.1	25.1	9.7	13.3
10/8	6	170.5	0.0704	0.189	37.6	37.8	14.5	20
10/8	8	227.3	0.0941	0.253	50.2	50.5	19.5	26.8
10/8	10	284.2	0.1178	0.253	50.5	50.6	19.4	26.9

^†^ CAP1298 and MPR121 values are dimensionless relative counts (engineering estimates from measured baseline-normalized responses), not directly comparable to the physical pF values of Σ–Δ devices. Selected configuration. ΔC plateaus at W = 8 mm; widening to 10 mm yields < 0.5% improvement while C_base increases ~25%.

**Table 4:** Species, body size, and mass of tested species.

Species	Body Length (mm)	Body Width (mm)	Mass (mg) Avg.	Cross-Section (mm^2^)
*Drosophila melanogaster*	2.5	1.0	1.0	0.79
*Aedes albopictus*	4.5	1.5	2.5	1.77
*Hasarius adansoni (Arachnid)*	6.0	3.5	20.0	9.62
*Bactrocera dorsalis*	8.0	3.0	15.0	7.07
*Chrysomya megacephala*	10.0	4.0	25.0	12.57
*Blattella germanica*	12.0	5.0	80.0	19.63

The six test species span approximately 25-fold in body cross-sectional area (0.79–19.63 mm^2^), covering the main body size range of common field pests.

**Table 5:** Sponge-based calibration metrics and verification results for four capacitive sensing ICs.

Calibration Item	Sponge Verification Result	Significance
Linearity	R^2^ > 0.99 (all 4 ICs achieved)	Linear relationship between ∆C and water content confirmed
Sensitivity consistency	6.14–6.47 fF/% (inter-IC difference < 6%)	All four ICs respond consistently to the same physical quantity
Repeatability	Σ–Δ CV < 2.5%, touch-type CV < 10%	Quantifies short-term noise level for each architecture
Volume independence	Identical ∆C across sponge sizes A/B/C	Capacitance depends on cross-sectional fill, not axial length

**Table 6:** Measurement repeatability across ICs (Sponge A, 40% water content, RH 40%).

IC	R^2^	Sensitivity (fF/%)	Mean CV (%)
FDC1004	0.9970	6.17	0.98
AD7746	0.9945	6.23	1.21
CAP1298	0.9878	6.27	6.61
MPR121	0.9953	6.56	4.34

**Table 7:** FDC1004 insect pass-through measurement statistical summary.

Species	Baseline Mean (pF)	Baseline SD (pF)	Events Detected	ΔC Mean (fF)	ΔC SD (fF)	SNR Mean	SNR SD
*Ae. albopictus*	0.5195	0.00525	5	17.54	0.54	3.34	0.1
*B. dorsalis*	0.6446	0.00362	25	18.15	4.38	5.01	1.21
*B. germanica*	0.4234	0.00275	28	16.72	5.96	6.08	2.17
*C. megacephala*	0.6507	0.00206	50	11.77	7.5	5.72	3.65
*D. melanogaster*	0.6442	0.00157	7	8.56	2.89	5.44	1.84
*H. adansoni*	0.4427	0.00521	15	18.26	1.98	3.5	0.38

**Table 8:** Baseline stability classification (CV%).

Level	Baseline CV (%)	Interpretation
Excellent	<0.5%	Corresponds to SNR > 200; high precision (upper range of analytical chemistry standards, RSD < 1%)
Good	0.5–1.0%	Corresponds to SNR 100–200; (RSD ≤ 2%)
Acceptable	1.0–2.0%	Corresponds to SNR 50–100; acceptable for most sensing applications
Insufficient	>2.0%	Corresponds to SNR < 50; baseline drift may cause false positives/negatives for small insect

**Table 9:** SNR_FR comparison of four ICs (RH 40%, 10 mm/s).

Species	Area (mm^2^)	FDC1004	AD7746	CAP1298	MPR121
		ΔC/SNR	ΔC/SNR	ΔC/SNR	ΔC/SNR
*D. melanogaster*	0.79	14.3/2.1	14.7/2.4	15.1/0.9	13.9/1.2
*Ae. albopictus*	1.77	31.4/4.7	31.0/5.0	32.5/2.0	31.9/2.7
*B. dorsalis*	7.07	151.9/22.5	154.7/25.3	153.2/9.3	156.3/13.2
*H. adansoni*	9.62	171.3/25.4	165.8/27.1	162.3/9.9	159.1/13.4
*C. megacephala*	12.57	234.2/34.7	238.8/39.1	239.4/14.6	233.5/19.7
*B. germanica*	19.63	351.5/52.1	347.7/56.7	361.3/21.9	353.0/29.7

**Table 10:** Coefficient of variation comparison between frozen specimens and live insects (CV%, averaged across IC × RH).

Species	Frozen CV (%)	Live CV (%)	ΔCV (pp)	Direction
*D. melanogaster*	5.39	5.44	+0.05	unstable
*Ae. albopictus*	5.28	5.49	+0.21	unstable
*H. adansoni*	5.44	5.39	−0.05	unstable
*B. dorsalis*	7.00	5.93	−1.07	Live more stable
*C. megacephala*	5.97	5.37	−0.60	Live more stable
*B. germanica*	5.88	7.45	+1.57	Frozen more stable

**Table 11:** Conversion factors for translating measurements obtained from frozen specimens to equivalent parameters in live insects.

Species	Body Length (mm)	Conversion Factor k	Recommendation
*D. melanogaster*	2.5	0.999	No correction needed
*Ae. albopictus*	4.5	0.998	No correction needed
*H. adansoni*	6	1.008	No correction needed
*B. dorsalis*	8	1.006	No correction needed
*C. megacephala*	10	1.016	Correction recommended
*B. germanica*	12	1.018	Correction recommended

## Data Availability

The data presented in this study are not publicly available due to intellectual property protection and technology transfer constraints associated with this work. To support reproducibility, the experimental procedures, system architecture, and data processing methods are described in sufficient detail in the manuscript. Representative results and key parameters are also provided to enable independent verification of the reported findings. Access to data is subject to institutional regulations and may be considered under specific conditions.

## Supplementary Materials

The following supporting information can be downloaded at: https://www.mdpi.com/article/10.3390/s26113306/s1. File S1. Detection Logic.

## Abbreviations

The following abbreviations are used in this manuscript:

aF	attofarad
CAPDAC	Capacitance Digital-to-Analog Converter
CDC	Capacitance-to-Digital Converter
CV	Coefficient of Variation
ΔC	Capacitance Change
ΔC_static	Static Capacitance Change
ΔC_transit	Transit Event Capacitance Change
fF	femtofarad
I^2^C	Inter-Integrated Circuit
ID	Inner Diameter
IQR	Interquartile Range
OD	Outer Diameter
pF	picofarad
R^2^	Coefficient of Determination
SD	Standard Deviation
SMBus	System Management Bus
SNR	Signal-to-Noise Ratio
SNR_FR	Field-relevant signal-to-noise estimate
SNR_IQR	IQR-based Signal-to-Noise Ratio
SNR_lab	Laboratory Signal-to-Noise Ratio
SPI	Serial Peripheral Interface
SPS	Samples Per Second
Σ-Δ	Sigma-Delta (modulation)
