# Supplementary material for: Capacitive Insect Sensing Under a Single Dual-Arc Geometry: A Laboratory Benchmark of Four CDC Architectures
Source: Sensors (Basel). 2026 May 22;26(11):3306. doi: 10.3390/s26113306 (PMC13259291; doi:10.3390/s26113306)
Supplement: Supplementary file 1 [file sensors-26-03306-s001.zip › sensors-4261004-supplementary.pdf]

Supplementary Materials:

S1. Detection Logic

```
#pseudo
INPUT: Capacitance signal C(t)

# Step 1: Baseline estimation
baseline = estimate_baseline(C)
sigma_noise = std(baseline)

# Step 2: Event extraction
event_segment = detect_transit(C)

# Step 3: Amplitude calculation
delta_C = peak(event_segment) - baseline_level

# Step 4: Detection rule
IF (delta_C / sigma_noise) >= gamma:
   event = "detected"
ELSE:
   event = " non-target"
```
